# Correlation of uric acid with body mass index based on NHANES 2013–2018 data: A cross-sectional study

**DOI:** 10.1097/MD.0000000000030646

**Published:** 2022-09-30

**Authors:** Huashuai Wang, Jia Yao, Ning Ding, Yongheng He

**Affiliations:** a Hunan University of Chinese Medicine, Hunan, China; b Department of Anorectal Surgery, Hunan Academy of Traditional Chinese Medicine Affiliated Hospital, Hunan, China; c School of Second Clinical Medicine, Guangzhou University of Chinese Medicine, Guangzhou, China; d Department of Endocrinology, Guangdong Provincial Hospital of Chinese Medicine, Guangzhou, China.

**Keywords:** body mass index, cross-sectional study, NHANES, uric acid

## Abstract

Clinical investigation of obesity-related risk factors aids in the early detection, prevention, and management of obesity. We aimed to examine the association between obesity and serum uric acid (sUA). A cross-sectional study was conducted including 18473 subjects from the National Health and Nutrition Examination Survey (NHANES). The exposure and outcome variables were sUA and body mass index (BMI), respectively. The weighted multivariate linear regression models and smooth curve fittings were conducted to assess the association between sUA and BMI. There were significantly positive correlations between sUA and BMI in both males and females (*β* = 1.414, 95% CI: 1.323−1.505, *P *< .0001, *β* = 1.853, 95% CI: 1.740−1.966, *P* < .0001, respectively). Furthermore, individuals in the higher sUA quartiles had higher BMI than those in the lowest quartile in both males and females. Subgroup analyses were stratified by race/ethnicity, results indicated the positive association of sUA with BMI in males remained in all races including Mexican American (*β* = 1.203, 95% CI: 0.965−1.442, *P *< .0001), other Hispanic (*β* = 1.126, 95% CI: 0.858−1.395, *P* < .0001), non-Hispanic White (*β* = 1.493, 95% CI: 1.343−1.642, *P *< .0001), non-Hispanic Black (*β* = 1.331, 95% CI: 1.122−1.540, *P *< .0001), and other races (*β* = 1.329, 95% CI: 1.115−1.544, *P* < .0001). And the positive association of sUA with BMI in females also remained in all races including Mexican American (*β* = 1.806, 95% CI: 1.520−2.092, *P* < .0001), other Hispanic (*β* = 2.033, 95% CI: 1.687−2.379, *P* < .0001), non-Hispanic White (*β* = 1.847, 95% CI: 1.657−2.037, *P* < .0001), non-Hispanic Black (*β* = 2.141, 95% CI: 1.874−2.408, *P* < .0001), and other races (*β* = 1.348, 95% CI: 1.081−1.615, *P* < .0001). The current cross-sectional study with 18473 US participants found that an elevated sUA was positively correlated with a higher BMI in males, females, and all kinds of races.

## 1. Introduction

Obesity is becoming a critical issue affecting individuals all over the world. Since the 1970s, global obesity has nearly quadrupled.^[1]^ Overweight adults accounted for more than 1.9 billion people (39% of the worldwide adult population) in 2016, with over 650 million obese.^[2]^ Obesity is linked to many comorbidities, including obstructive sleep apnea, a prothrombotic condition, dyslipidemia, diabetes, hypertension, metabolic syndrome, and cardiovascular disease.^[3,4]^ Obesity is one of the most serious public health issues, putting a huge strain on affected individuals, healthcare institutions, and society as a whole.^[5]^

Clinical investigation of obesity-related risk factors aids in the early detection, prevention, and management of obesity. As a result, ongoing research is examining the relationship between obesity and serum uric acid (sUA). UA is a byproduct of purine metabolism and hyperuricemia results from an imbalance in uric acid synthesis and excretion. A growing body of research suggests that sUA levels are linked to metabolic and cardiovascular disorders.^[6–9]^ Furthermore, some investigations have found a relationship between obesity and hyperuricemia.^[10–14]^ Elevated sUA and obesity are likely to interact through numerous pathways, elevated sUA can speed up hepatic and peripheral lipogenesis thus causing obesity.^[15]^ The National Health and Nutrition Examination Survey (NHANES) is a key initiative of the National Center for Health Data in the United States (US), which is part of the Centers for Disease Control and Prevention and is in charge of producing essential and medical statistical data for the whole nation. Nevertheless, based on NHANES data, there is a scarcity of research on the correlation between sUA and obesity.

Given the probable association between sUA and obesity, and their role in the occurrence and development of cardiovascular diseases and raised mortality, a greater understanding of the interaction of sUA with obesity is required. Based on the 2013 to 2018 NHANES data, this study aimed to assess the correlation between sUA and obesity.

## 2. Materials and Methods

### 2.1. Study population

The NHANES is a typical survey including the national subjects in the US and provides a plethora of data about the nutrition and health of the general US population by employing a complex and multistage sampling technique. The study was granted ethical approval by the National Center for Health Statistics (NCHS) in the US. All methods were performed following the relevant guidelines and regulations. The written informed consents were obtained from all participants or their proxies.

The present study included NHANES data from 2013 to 2018 (n = 29400), which represented 3 cycles. After excluding individuals with missing sUA (n = 10694) and body mass index (BMI) (n = 3584), 18,473 subjects were finally included in this study (Figure S1, Supplemental Digital Content 1, http://links.lww.com/MD/H343).

### 2.2. Measurements of covariates

The exposure variable was sUA (mg/dL). The Roche Cobas 6000 (c501 module) technique was used to measure sUA. BMI is an important index for diagnosing obesity, and the outcome variable was BMI, which was calculated using the formula of weight (kg)/height (m)^2^.

Categorical variables of covariates included in our analysis were as follows: gender (male or female), race/ethnicity (Mexican American, other Hispanic, non-Hispanic white, non-Hispanic black, or other race), education levels (less than high school, high school, or more than high school), marital status (living with a spouse or partner: yes or no), alcohol consumption (yes or no), and smoking behavior (yes or no). Continuous covariates: age (years), energy intake (kcal), poverty to income ratio, minutes sedentary activity (minutes), total cholesterol (TC) (mg/dL), triglyceride (mg/dL), high-density lipoprotein cholesterol (HDL-C) (mg/dL), low-density lipoprotein cholesterol (LDL-C) (mg/dL), fasting blood glucose (mg/dL), glycohemoglobin (%), systolic blood pressure (SBP) (mm Hg), and diastolic blood pressure (DBP) (mm Hg). The criteria for selecting covariates were based on previously published research and variables.^[16]^ The complete data on sUA, BMI, and confounders can be found at http://www.cdc.gov/nchs/nhanes/.

### 2.3. Statistical analysis

To account for the significant volatility in our data set, we utilized a weighted and variance estimation strategy. To assess the correlation of sUA with BMI, a weighted multivariate logistic regression model was utilized. To count the discrepancies between subgroups, we applied the weighted *χ*^2^ test for the categorical data and the weighted linear regression model for the continuous variables. The stratified multivariate regression analysis was used to accomplish the subgroup analysis. Additionally, smooth curve fittings and generalized additive models were applied to clarify the non-linear relation between sUA and BMI. When nonlinearity was identified, the inflection point in the connection between sUA and BMI was estimated by a recursive technique, and a two-piecewise linear regression model was performed on both sides of the inflection point. Statistical analyses were carried out using the R package (http://www.r-project.org) and EmpowerStats (http://www.empowerstats.com), with a *P* < .05 threshold deemed statistically significant.

## 3. Results

In our research, 18,473 subjects were included. Among them, 8953 (48.47%) subjects were males, 9520 (51.53%) were females. In males, the weighted characteristics were subclassified according to sUA quartiles (Q1: 1.5–5.1 mg/dL; Q2: 5.2–5.9 mg/dL; Q3: 6.0–6.8 mg/dL; and Q4: 6.9–15.1 mg/dL), as presented in Table 1. Except for minutes of sedentary activity, there were significant differences in baseline characteristics between the different sUA quartiles. Individuals in the highest sUA quartile were more prone to be with higher age, BMI, TC, triglyceride, LDL-C, SBP, and DBP. In addition, participants with the greatest sUA levels had reduced HDL-C levels. In females, the weighted characteristics were subclassified according to sUA quartiles (Q1: 0.7–3.9 mg/dL; Q2: 4.0–4.6 mg/dL; Q3: 4.7–5.5 mg/dL; and Q4: 5.6–18.0 mg/dL), as showed in Table 2. There were significant differences in baseline characteristics between the different sUA quartiles. Individuals in the highest sUA quartile were more prone to be with higher age, BMI, glycohemoglobin, TC, LDL-C, SBP, and DBP. In addition, subjects with the greatest sUA levels had reduced HDL-C levels.

**Table 1:** Weighted characteristics in male subjects based on serum uric acid quartiles.

Serum uric acid (mg/dL)	Q1 (1.5−5.1)	Q2 (5.2−5.9)	Q3 (6.0−6.8)	Q4 (6.9−15.1)	*P* value
Age (yr)	42.35 ± 20.08	42.65 ± 18.91	43.03 ± 18.62	45.07 ± 18.32	<.0001
Race/ethnicity (%)					.0002
Mexican American	11.74	10.89	10.53	8.29	
Other Hispanic	6.90	6.00	6.77	5.87	
Non-Hispanic White	61.01	65.31	63.21	64.46	
Non-Hispanic Black	11.85	9.18	9.37	11.08	
Other races	8.49	8.62	10.12	10.29	
Alcohol consumption (%)					
<12 drinks daily	55.28	67.84	63.77	68.53	
≥12 drinks daily	1.21	1.98	2.09	2.35	
Unknown	43.50	30.17	34.14	29.12	
Smoking behavior (%)					<.0001
Smoked at least 100 cigarettes in life	45.64	42.47	44.36	47.37	
Smoked less than 100 cigarettes in life	39.19	47.44	47.16	47.06	
Unknown	15.17	10.09	8.48	5.57	
Education level (%)					<.0001
Less than high school	13.40	12.33	12.34	12.15	
High school	20.68	20.24	20.62	24.02	
More than high school	48.52	53.98	54.99	56.03	
Unknown	17.40	13.45	12.05	7.79	
Marital status (%)					<.0001
Live with a partner	54.44	60.15	60.52	60.96	
Live alone	28.16	26.45	27.46	31.24	
Unknown	17.41	13.40	12.02	7.80	
Ratio of family income to poverty	2.82 ± 1.57	3.04 ± 1.57	2.98 ± 1.60	3.07 ± 1.59	<.0001
Energy (kcal)	2520.70 ± 1031.93	2510.61 ± 1030.16	2425.80 ± 1053.41	2406.84 ± 1050.06	.0001
Minutes sedentary activity (minutes)	423.18 ± 591.99	423.15 ± 461.95	410.01 ± 449.81	450.75 ± 600.26	.0634
Body mass index (kg/m^2^)	26.02 ± 5.84	27.38 ± 5.73	28.84 ± 5.83	31.73 ± 7.05	<.0001
Glycohemoglobin (%)	5.77 ± 1.24	5.57 ± 0.89	5.54 ± 0.81	5.66 ± 0.85	<.0001
Fasting blood glucose (mg/dL)	112.05 ± 33.06	108.00 ± 19.08	107.95 ± 17.94	110.24 ± 19.94	<.0001
Total cholesterol (mg/dL)	174.10 ± 39.80	180.61 ± 42.39	184.69 ± 40.40	189.94 ± 42.45	<.0001
Triglyceride (mg/dL)	105.11 ± 59.27	108.02 ± 58.98	114.72 ± 68.97	128.86 ± 98.73	<.0001
High-density lipoprotein cholesterol (mg/dL)	50.86 ± 15.31	49.67 ± 13.86	47.89 ± 13.73	45.80 ± 13.08	<.0001
Low-density lipoprotein cholesterol (mg/dL)	104.81 ± 23.89	106.80 ± 23.76	108.59 ± 23.01	110.30 ± 24.36	<.0001
Systolic blood pressure (mm Hg)	121.17 ± 14.98	121.54 ± 14.62	122.18 ± 14.67	125.01 ± 15.68	<.0001
Diastolic blood pressure (mm Hg)	68.93 ± 12.98	69.88 ± 12.88	70.15 ± 12.94	72.49 ± 12.60	<.0001

Mean ± SD for continuous variables: the *P* value was calculated by the weighted linear regression model. (%) for categorical variables: the *P* value was calculated by the weighted chi-square test.

**Table 2:** Weighted characteristics in female subjects based on serum uric acid quartiles.

Serum uric acid (mg/dL)	Q1 (0.7−3.9)	Q2 (4.0−4.6)	Q3 (4.7−5.5)	Q4 (5.6−18.0)	*P* value
Age (yr)	40.50 ± 17.53	42.32 ± 18.81	44.86 ± 19.19	51.72 ± 19.70	<.0001
Race/ethnicity (%)					<.0001
Mexican American	11.55	10.40	8.27	7.31	
Other Hispanic	8.08	7.62	5.95	4.80	
Non-Hispanic White	60.84	60.97	64.99	65.16	
Non-Hispanic Black	12.27	10.36	10.69	13.54	
Other races	7.26	10.66	10.10	9.19	
Alcohol consumption (%)					.0127
<12 drinks daily	59.72	59.95	63.17	57.78	
≥12 drinks daily	0.29	0.26	0.30	0.24	
Unknown	39.99	39.79	36.53	41.98	
Smoking behavior (%)					<.0001
Smoked at least 100 cigarettes in life	29.54	30.96	31.66	37.10	
Smoked less than 100 cigarettes in life	59.38	59.03	60.71	57.59	
Unknown	11.08	10.01	7.63	5.32	
Education level (%)					<.0001
Less than high school	11.56	10.70	10.92	12.45	
High school	17.28	18.09	19.89	22.90	
More than high school	57.17	58.29	58.99	57.53	
Unknown	13.99	12.92	10.20	7.11	
Marital status (%)					<.0001
Live with a partner	54.60	56.65	52.06	49.94	
Live alone	31.41	30.40	37.87	42.99	
Unknown	13.99	12.95	10.07	7.07	
Ratio of family income to poverty	2.87 ± 1.60	2.91 ± 1.62	2.87 ± 1.59	2.67 ± 1.56	<.0001
Energy (kcal)	1932.45 ± 759.49	1871.57 ± 755.43	1862.06 ± 746.47	1766.98 ± 715.70	<.0001
Minutes sedentary activity (minutes)	419.54 ± 645.28	444.47 ± 696.65	438.51 ± 649.12	521.26 ± 1039.42	<.0001
Body mass index (kg/m^2^)	25.76 ± 5.79	27.61 ± 6.79	29.59 ± 7.54	33.43 ± 8.60	<.0001
Glycohemoglobin (%)	5.48 ± 0.93	5.48 ± 0.78	5.54 ± 0.73	5.81 ± 0.97	<.0001
Fasting blood glucose (mg/dL)	105.72 ± 20.53	105.26 ± 17.26	106.06 ± 16.97	109.73 ± 22.83	<.0001
Total cholesterol (mg/dL)	183.44 ± 39.74	187.46 ± 39.03	190.68 ± 41.25	196.74 ± 42.54	<.0001
Triglyceride (mg/dL)	99.80 ± 89.49	99.73 ± 37.73	105.14 ± 45.42	117.41 ± 52.08	<.0001
High-density lipoprotein cholesterol (mg/dL)	62.31 ± 16.52	60.64 ± 17.38	58.35 ± 16.57	54.91 ± 16.40	<.0001
Low-density lipoprotein cholesterol (mg/dL)	105.28 ± 20.98	106.79 ± 22.33	108.73 ± 24.51	110.94 ± 27.49	<.0001
Systolic blood pressure (mm Hg)	115.53 ± 15.85	117.55 ± 16.44	120.43 ± 17.06	125.16 ± 18.51	<.0001
Diastolic blood pressure (mm Hg)	67.41 ± 10.91	68.10 ± 11.52	68.66 ± 12.00	69.24 ± 11.96	<.0001

Mean ± SD for continuous variables: the *P* value was calculated by the weighted linear regression model. (%) for categorical variables: the *P* value was calculated by the weighted chi-square test.

Table 3 showed the findings of the multivariate regression analysis in males. SUA was positively correlated with BMI in the unadjusted model (*β* = 1.741, 95% CI: 1.642–1.840, *P* < .0001). After adjusting for covariates, this significant link remained in model 2 (*β* = 1.716, 95% CI: 1.619–1.813, *P* < .0001) and 3 (*β* = 1.414, 95% CI: 1.323–1.505, *P* < .0001). Furthermore, individuals in the higher sUA quartiles (5.2–5.9 mg/dL, 6.0–6.8 mg/dL, and 6.9–15.1 mg/dL) had higher BMI than those in the lowest quartile (1.5–5.1 mg/dL) after alternating the sUA from a continuous variable to a categorical variable (*β* = 1.153, 95% CI: 0.825–1.481, *P* < .0001; *β* = 2.376, 95% CI: 2.049–2.703, *P* < .0001; *β* = 4.566, 95% CI: 4.232–4.900, *P* < .0001, respectively).

**Table 3:** The association between serum uric acid (mg/dL) and body mass index (BMI) (kg/m^2^) in male.

	Model 1 *β* (95% CI) *P* value	Model 2 *β* (95% CI) *P* value	Model 3 *β* (95% CI) *P* value
Serum uric acid (mg/dL)	1.741 (1.642, 1.840) <.00001	1.716 (1.619, 1.813) <.00001	1.414 (1.323, 1.505) <.00001
Serum uric acid categories			
Q1 (1.5−5.1 mg/dL)	Reference	Reference	Reference
Q2 (5.2−5.9 mg/dL)	1.360 (0.993, 1.728) <.00001	1.364 (1.005, 1.723) <.00001	1.153 (0.825, 1.481) <.00001
Q3 (6.0−6.8 mg/dL)	2.815 (2.451, 3.179) <.00001	2.818 (2.462, 3.174) <.00001	2.376 (2.049, 2.703) <.00001
Q4 (6.9−15.1 mg/dL)	5.705 (5.339, 6.071) <.00001	5.621 (5.262, 5.980) <.00001	4.566 (4.232, 4.900) <.00001
Subgroup analysis stratified by race/ethnicity			
Mexican American	1.662 (1.411, 1.912) <.00001	1.663 (1.419, 1.908) <.00001	1.203 (0.965, 1.442) <.00001
Other Hispanic	1.533 (1.239, 1.827) <.00001	1.438 (1.148, 1.728) <.00001	1.126 (0.858, 1.395) <.00001
Non-Hispanic White	1.814 (1.650, 1.979) <.00001	1.773 (1.612, 1.933) <.00001	1.493 (1.343, 1.642) <.00001
Non-Hispanic Black	1.877 (1.657, 2.097) <.00001	1.765 (1.542, 1.988) <.00001	1.331 (1.122, 1.540) <.00001
Other races	1.521 (1.294, 1.749) <.00001	1.551 (1.324, 1.777) <.00001	1.329 (1.115, 1.544) <.00001

Model 1: no covariates were adjusted. Model 2: age and race/ethnicity were adjusted. Model 3: age, race/ethnicity, alcohol consumption, smoking behavior, education level, marital status, ratio of family income to poverty, energy, minutes sedentary activity, glycohemoglobin, fasting glucose, total cholesterol, triglyceride, high-density lipoprotein cholesterol, low-density lipoprotein cholesterol, systolic blood pressure, and diastolic blood pressure were adjusted. In the subgroup analysis stratified by race/ethnicity; the model is not adjusted for race/ethnicity.

According to the results of subgroup analyses stratified by race, the positive association of sUA with BMI remained in all races including Mexican American (*β* = 1.203, 95% CI: 0.965–1.442, *P *< .0001), other Hispanic (*β* = 1.126, 95% CI: 0.858–1.395, *P* < .0001), non-Hispanic White (*β* = 1.493, 95% CI: 1.343–1.642, *P* < .0001), non-Hispanic Black (*β* = 1.331, 95% CI: 1.122–1.540, *P* < .0001), and other races (*β* = 1.329, 95% CI: 1.115–1.544, *P* < .0001).

**Figure 1.**
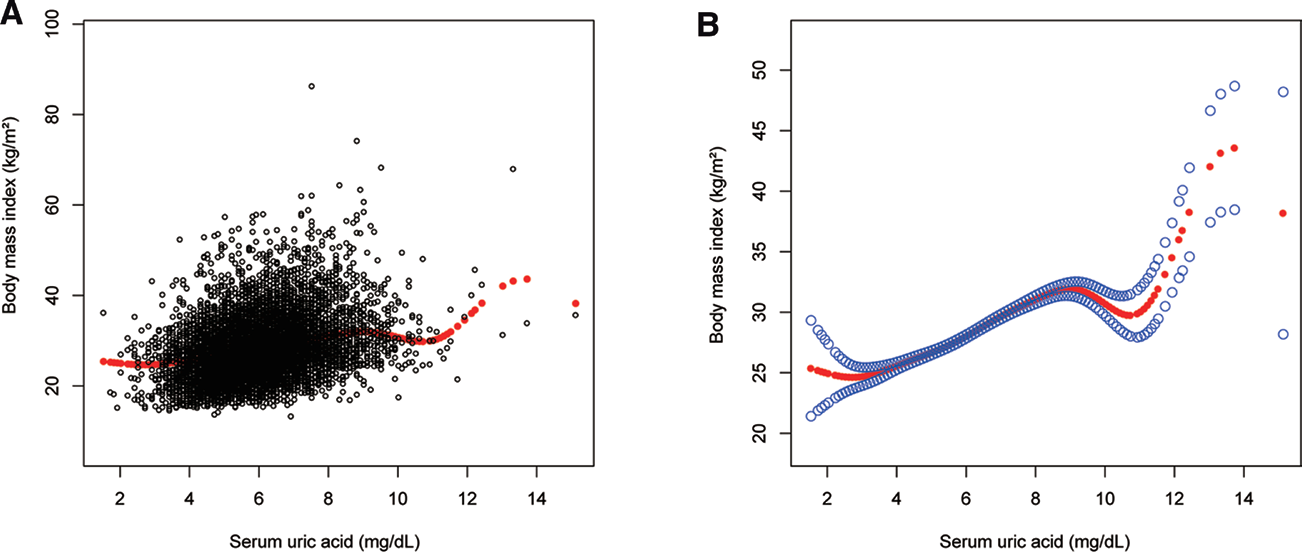
The association between serum uric acid and body mass index in males. (A) Each black point represents a sample. (B) The solid red line represents the smooth curve fit between variables. Blue bands represent the 95% of confidence interval from the fit. Age, race/ethnicity, alcohol consumption, smoking behavior, education level, marital status, the ratio of family income to poverty, energy, minutes of sedentary activity, glycohemoglobin, fasting glucose, total cholesterol, triglyceride, high-density lipoprotein cholesterol, low-density lipoprotein cholesterol, systolic blood pressure, and diastolic blood pressure were adjusted.

**Figure 2.**
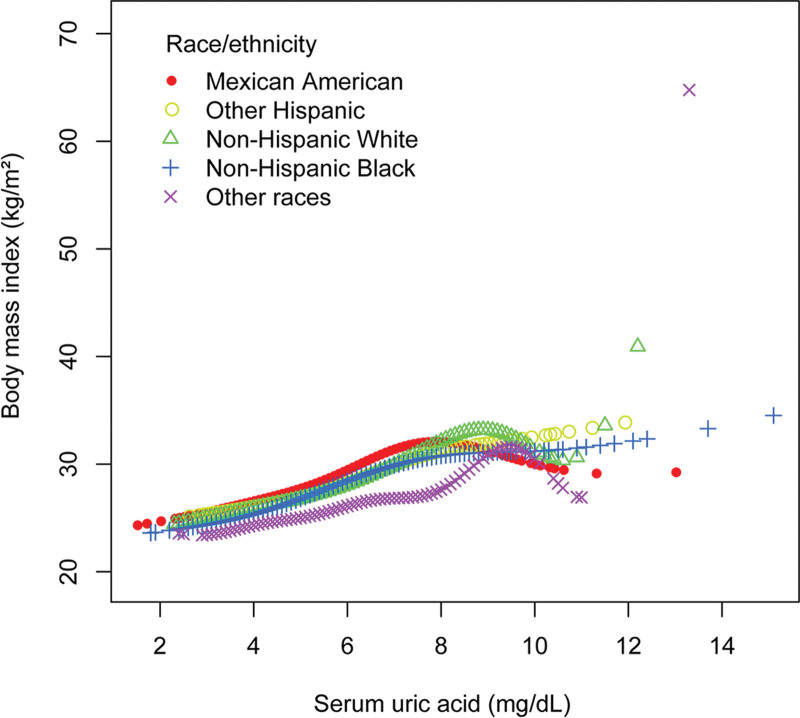
The association of serum uric acid with body mass index in males stratified by race/ethnicity. Age, alcohol consumption, smoking behavior, education level, marital status, the ratio of family income to poverty, energy, minutes of sedentary activity, glycohemoglobin, fasting glucose, total cholesterol, triglyceride, high-density lipoprotein cholesterol, low-density lipoprotein cholesterol, systolic blood pressure, and diastolic blood pressure were adjusted.

To evaluate the nonlinear connection between sUA and BMI in males and stratified by race/ethnicity, smooth curve fittings, and generalized additive models were applied (Figs. 1 and 2). The association between sUA and BMI in males (turning points: 2.6, 9.0, and 10.5 mg/dL) followed an inverted U-shaped curve (Table 4).

**Table 4:** Threshold effect analysis of serum uric acid (mg/dL) on BMI (kg/m^2^) in male using the two-piecewise linear regression model.

Serum uric acid (mg/dL)	Adjusted *β* (95% CI), *P* value
Fitting by the standard linear model	1.414 (1.323, 1.505) <.0001
Fitting by the two-piecewise linear model	
Inflection point	2.6, 9.0, 10.5
<2.6	−4.267 (−10.925, 2.390) 0.2091
2.6−9.0	1.422 (1.330, 1.513) <.0001
9.0−10.5	−1.441 (−2.949, 0.067) 0.0611
>10.5	6.902 (4.115, 9.689) <.0001
Log likelihood ratio	<.001

Age, race/ethnicity, alcohol consumption, smoking behavior, education level, marital status, ratio of family income to poverty, energy, minutes sedentary activity, glycohemoglobin, fasting glucose, total cholesterol, triglyceride, high-density lipoprotein cholesterol, low-density lipoprotein cholesterol, systolic blood pressure, and diastolic blood pressure were adjusted

**Figure 3.**
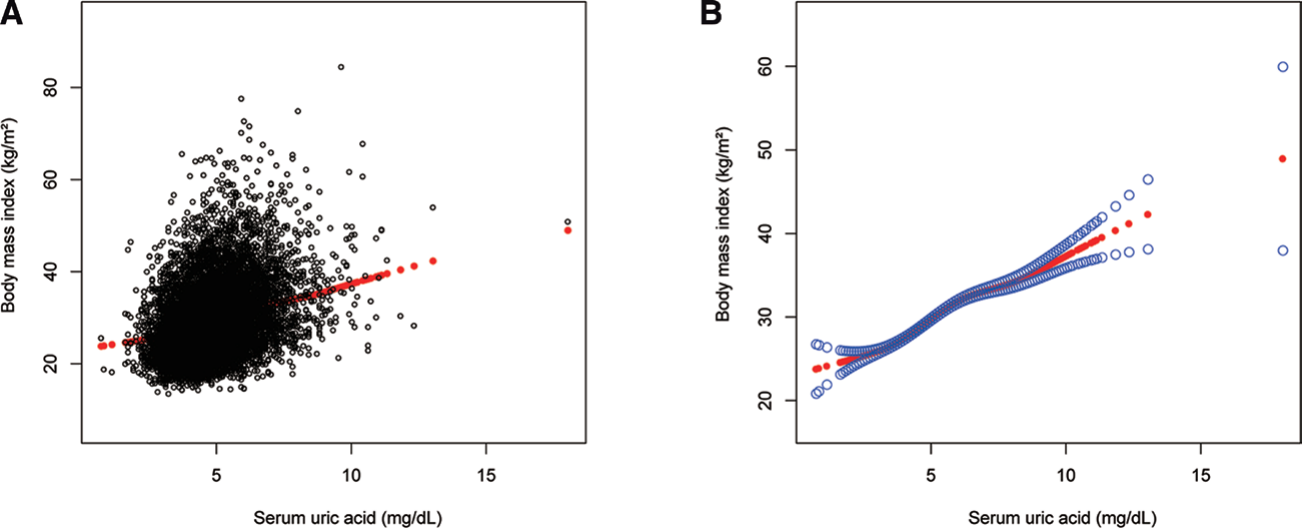
The association between serum uric acid and body mass index in females. (A) Each black point represents a sample. (B) The solid red line represents the smooth curve fit between variables. Blue bands represent the 95% of confidence interval from the fit. Age, race/ethnicity, alcohol consumption, smoking behavior, education level, marital status, the ratio of family income to poverty, energy, minutes of sedentary activity, glycohemoglobin, fasting glucose, total cholesterol, triglyceride, high-density lipoprotein cholesterol, low-density lipoprotein cholesterol, systolic blood pressure, and diastolic blood pressure were adjusted.

**Figure 4.**
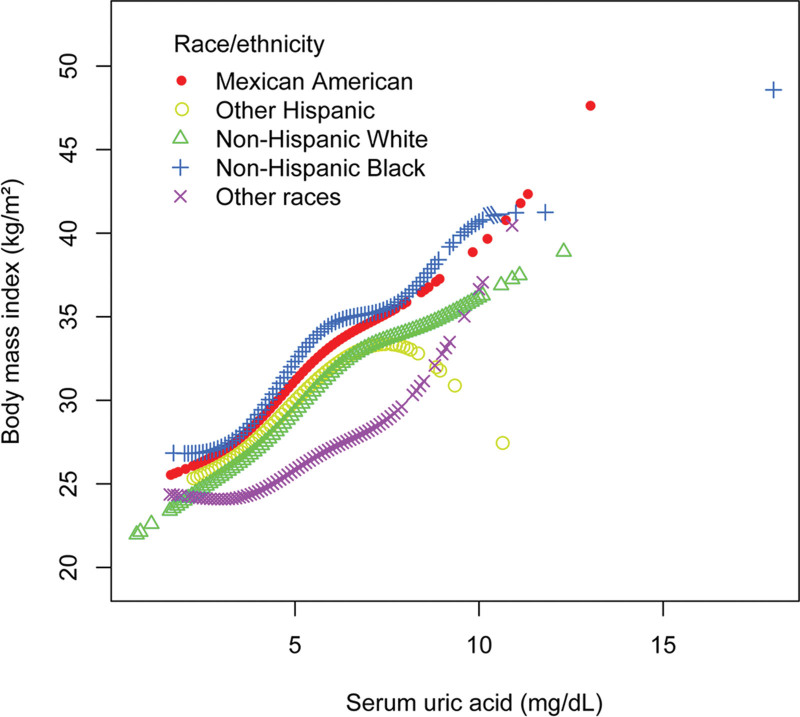
The association of serum uric acid with body mass index in females stratified by race/ethnicity. Age, alcohol consumption, smoking behavior, education level, marital status, the ratio of family income to poverty, energy, minutes of sedentary activity, glycohemoglobin, fasting glucose, total cholesterol, triglyceride, high-density lipoprotein cholesterol, low-density lipoprotein cholesterol, systolic blood pressure, and diastolic blood pressure were adjusted.

Table 5 showed the findings of the multivariate regression analysis in females. SUA was also positively correlated with BMI in females in the unadjusted model (*β* = 2.330, 95% CI: 2.212–2.448, *P *< .0001). After adjusting for covariates, this significant link remained in model 2 (*β* = 2.260, 95% CI: 2.140–2.379, *P* < .0001) and 3 (*β* = 1.853, 95% CI: 1.740–1.966, *P* < .0001). Furthermore, individuals in the higher sUA quartiles (4.0–4.6, 4.7–5.5, and 5.6–18.0 mg/dL) had higher BMI than those in the lower quartile (0.7 to 3.9 mg/dL) after alternating the sUA from a continuous variable to a categorical variable (*β* = 1.702, 95% CI: 1.320–2.084, *P* < .0001; *β* = 3.182, 95% CI: 2.810–3.555, *P* < .0001; *β* = 6.078, 95% CI: 5.681–6.475, *P* < .0001, respectively). According to the results of subgroup analyses stratified by race, the positive association of sUA with BMI in females remained in all races including Mexican American (*β* = 1.806, 95% CI: 1.520–2.092, *P* < .0001), other Hispanic (*β* = 2.033, 95% CI: 1.687–2.379, *P* < .0001), non-Hispanic White (*β* = 1.847, 95% CI: 1.657–2.037, *P* < .0001), non-Hispanic Black (*β* = 2.141, 95% CI: 1.874–2.408, *P* < .0001), and other races (*β* = 1.348, 95% CI: 1.081–1.615, *P* < .0001). Moreover, to evaluate the nonlinear connection between sUA and BMI in females and stratified by race/ethnicity, smooth curve fittings and generalized additive models were applied (Figs. 3 and 4). The association between sUA and BMI in females showed a turning point of 6.1 mg/dL (Table 6).

**Table 5:** The association between serum uric acid (mg/dL) and BMI (kg/m^2^) in female.

	Model 1 *β* (95% CI) *P* value	Model 2 *β* (95% CI) *P* value	Model 3 *β* (95% CI) *P* value
Serum uric acid (mg/dL)	2.330 (2.212, 2.448) <.00001	2.260 (2.140, 2.379) <.00001	1.853 (1.740, 1.966) <.00001
Serum uric acid categories			
Q1 (0.7−3.9 mg/dL)	Reference	Reference	Reference
Q2 (4.0−4.6 mg/dL)	1.853 (1.429, 2.277) <.00001	1.958 (1.542, 2.375) <.00001	1.702 (1.320, 2.084) <.00001
Q3 (4.7−5.5 mg/dL)	3.833 (3.426, 4.241) <.00001	3.906 (3.504, 4.308) <.00001	3.182 (2.810, 3.555) <.00001
Q4 (5.6−18.0 mg/dL)	7.673 (7.255, 8.091) <.00001	7.475 (7.055, 7.895) <.00001	6.078 (5.681, 6.475) <.00001
Subgroup analysis stratified by race/ethnicity			
Mexican American	2.355 (2.055, 2.654) <.00001	2.191 (1.891, 2.491) <.00001	1.806 (1.520, 2.092) <.00001
Other Hispanic	2.585 (2.236, 2.935) <.00001	2.456 (2.100, 2.811) <.00001	2.033 (1.687, 2.379) <.00001
Non-Hispanic White	2.353 (2.156, 2.550) <.00001	2.275 (2.073, 2.477) <.00001	1.847 (1.657, 2.037) <.00001
Non-Hispanic Black	2.431 (2.180, 2.683) <.00001	2.386 (2.114, 2.659) <.00001	2.141 (1.874, 2.408) <.00001
Other races	2.006 (1.723, 2.289) <.00001	1.957 (1.668, 2.246) <.00001	1.348 (1.081, 1.615) <.00001

Model 1: no covariates were adjusted. Model 2: age and race/ethnicity were adjusted. Model 3: age, race/ethnicity, alcohol consumption, smoking behavior, education level, marital status, ratio of family income to poverty, energy, minutes sedentary activity, glycohemoglobin, fasting glucose, total cholesterol, triglyceride, high-density lipoprotein cholesterol, low-density lipoprotein cholesterol, systolic blood pressure, and diastolic blood pressure were adjusted. In the subgroup analysis stratified by race/ethnicity; the model is not adjusted for race/ethnicity.

**Table 6:** Threshold effect analysis of serum uric acid (mg/dL) on BMI (kg/m^2^) in female using the two-piecewise linear regression model.

Serum uric acid (mg/dL)	Adjusted *β* (95% CI), *P* value
Fitting by the standard linear model	1.853 (1.740, 1.966) <.0001
Fitting by the two-piecewise linear model	
Inflection point	6.1
<6.1	2.077 (1.929, 2.225) <.0001
>6.1	1.129 (0.798, 1.459) <.0001
Log likelihood ratio	<.001

Age, race/ethnicity, alcohol consumption, smoking behavior, education level, marital status, ratio of family income to poverty, energy, minutes sedentary activity, glycohemoglobin, fasting glucose, total cholesterol, triglyceride, high-density lipoprotein cholesterol, low-density lipoprotein cholesterol, systolic blood pressure, and diastolic blood pressure were adjusted

## 4. Discussion

The current study of a nationally representative sample of 18473 US participants found that an elevated sUA was positively associated with a higher BMI in both males and females, and all races including Mexican Americans, other Hispanics, non-Hispanic white, non-Hispanic black, and other races.

Currently, obesity and hyperuricemia, along with their related health issues, have developed as serious public health concerns as their growing prevalence. Obesity and sUA coexistence may accelerate disease progression, resulting in a higher medical and economic burden, posing additional difficulties to chronic disease prevention and treatment. Although changes in obesity have been observed to be separately linked with changes in sUA content, previous pathophysiological and metabolic research have just reported that they may interact. In participants from the US, elevated sUA levels were positively connected with higher BMI in both males and females, according to our current findings. This is consistent with earlier epidemiological and clinical evidence of a substantial significant positive connection between sUA and obesity in adult Chinese, Japanese, Indian, Pakistani, Iraqi, and Bangladeshi populations.^[17–19]^ The link between sUA and obesity can be explained by several mechanisms. Obesity or extra body fat may be linked with elevated sUS production and insufficient excretion due to insulin resistance, resulting in impaired UA metabolism and even hyperuricemia.^[14]^ Meanwhile, sUA has the potential to cause obesity by boosting the production of liver and peripheral fat,^[15]^ thus, forming a vicious cycle of hyperuricemia-obesity. Moreover, dysfunctions in glycolipid and UA metabolism may both enhance the cohabitation of the two components. As a result of the intimate biological association between sUA and BMI, it is critical for preventive medicine to closely evaluate the interplay between sUA and BMI.

Moreover, our findings revealed a considerable positive correlation of sUA levels with BMI in both men and women, however, it may be stronger in women. We further evaluated the nonlinear connection between sUA and BMI. The association between sUA and BMI in males (turning points: 2.6, 9.0, and 10.5 mg/dL) followed an inverted U-shaped curve. And the association between sUA and BMI in females showed a turning point of 6.1 mg/dL. It can be seen that different uric acid levels have different effects on BMI, and there are gender differences. This is similar to the study that found a positive correlation between BMI and sUA in healthy Chinese people and revealed that the related risk of sUA levels and obesity was greater in women.^[20]^ Another Thailand research also reported high sUA levels were correlated with a greater risk of obesity in women.^[21]^ Women with obesity, according to Kim et al, had a greater risk of severe hyperuricemia than males.^[10,11]^ Several studies^[22–24]^ reported that possible mechanisms contained hormonal impact or sex variations in insulin sensitivity and body fat composition. In contrast to our findings, research in Bangladesh and Japan found that greater sUA correlated with obesity in males more strongly.^[25,26]^ Furthermore, studies have suggested sex-based differences in many diseases. Although age and potential variables were adjusted for the multivariate regression analysis in the present analysis, the sex-based differences we recognized could also be influenced by unmeasured confounding variables, considering the baseline discrepancy in lifestyle and social aspects, such as smoking, alcohol drinking, and education levels between men and women. Taken as a whole, it makes sense to clarify the differences in the relations between sUA concentrations and obesity in men and women. Clinical recommendations should pay more attention to the gender disparity. More investigations on the physiology of sex in connection to sUA and obesity are needed.

The current study’s limitations mainly include its cross-sectional methodology, which cannot support a causal association between sUA and obesity. Secondly, further fundamental mechanistic investigations and large-sample prospective research are needed to determine the precise mechanism of the relationship between sUA and obesity. Thirdly, we were unable to obtain more detailed data since this survey did not include questions concerning gout diagnosis or medication use, such as urate-lowering drugs and other medicines that might alter sUA levels and body weight. Fourthly, the risk of bias due to other potential variables that we could not account for remains. Despite its limitations, the findings might be helpful for public health because the positive correlation between sUA levels and obesity was identified based on a large nationally representative survey database in the present study.

## 5. Conclusion

The current cross-sectional study with 18,473 US participants showed that an elevated sUA was positively connected with a higher BMI in both men and women, and all races including Mexican Americans, other Hispanics, non-Hispanic white, non-Hispanic black, and other races.

## Author contributions

**Conceptualization:** Huashuai Wang, Jia Yao.

**Data curation:** Jia Yao, Ning Ding.

**Formal analysis:** Huashuai Wang.

**Project administration:** Yongheng He.

**Supervision:** Yongheng He.

**Validation:** Jia Yao, Yongheng He.

**Writing – original draft:** Huashuai Wang, Jia Yao.

**Writing – review & editing:** Yongheng He.

**HW and JY contributed equally to this work.**

## Supplementary Material


