# Supplementary figures and images for: Correlation of uric acid with body mass index based on NHANES 2013–2018 data: A cross-sectional study
Source: Medicine (Baltimore). 2022 Sep 30;101(39):e30646. doi: 10.1097/MD.0000000000030646 (PMC9524866; doi:10.1097/MD.0000000000030646)

### Supplementary Figure

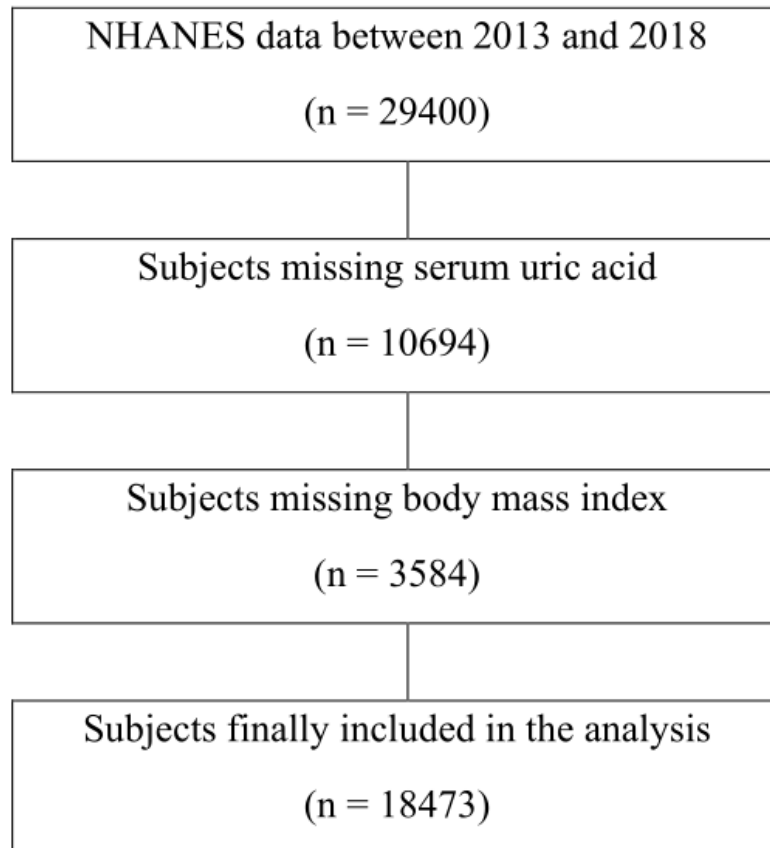

Supplementary Figure 1. Study flow diagram.

Supplement: Supplementary file 1 [file medi-101-e30646-s001.pdf]
